# Dynamics of colistin and tobramycin resistance among *Enterobacter cloacae* during prolonged use of selective decontamination of the digestive tract

**DOI:** 10.1186/s13756-018-0356-7

**Published:** 2018-05-22

**Authors:** M. J. D. Dautzenberg, J. R. Bayjanov, M. A. Leverstein-van Hall, A. E. Muller, L. B. S. Gelinck, C. L. Jansen, E. M. S. Leyten, T. Ruys, J. Scharringa, R. E. van der Starre, A. C. Fluit, M. J. M. Bonten

**Affiliations:** 1 Department of Medical Microbiology, University Medical Center Utrecht, Utrecht, the Netherlands; 2 Julius Center for Health Sciences and Primary Care, University Medical Center Utrecht, Utrecht, the Netherlands; 3 Department of Medical Microbiology, Radboud University Medical Center, Nijmegen, the Netherlands; 4 Department of Medical Microbiology, Haaglanden Medisch Centrum, The Hague, the Netherlands; 5 Department of Medical Microbiology, Alrijne Hospital, Leiden, the Netherlands; 6 Department of Internal Medicine, Haaglanden Medisch Centrum, The Hague, the Netherlands; 7 Department of Intensive Care Medicine, Haaglanden Medisch Centrum, The Hague, the Netherlands

**Keywords:** Colistin resistance, Tobramycin resistance, *Enterobacter cloacae*, Intensive care unit, Selective digestive tract decontamination

## Abstract

**Background:**

A high prevalence of colistin resistance among *E. cloacae* isolates in two intensive care units (ICU) (of 16 and 6 beds) using selective digestive decontamination (SDD) since 1990 instigated a retrospective and prospective investigation to quantify the role of clonal transmission. SDD is topical application of colistin and tobramycin and systemic use of cefotaxime during the first days of ICU-admission.

**Methods:**

Multi-resistant *E. cloacae* (MREb) was defined as ESBL production and/or tobramycin non-susceptibility and/or colistin non-susceptibility. Incidence of acquisition and prevalence of carriage with MREb was determined from microbiological culture results.

**Results:**

Colistin-resistant *E. cloacae* was first detected in November 2009 and carriage was demonstrated in 141 patients until October 2014. Mean incidence of MREb acquisition was 4.61 and 1.86 per 1000 days at risk in ICUs 1 and 2, respectively, and the mean monthly prevalence of MREb in both ICUs was 7.0 and 3.1%, respectively, without a discernible trend in time. Conversion rates from carriage of colistin-susceptible to resistant *E. cloacae* were 0.20 and 0.13 per 1000 patient days, respectively. Whole genome sequencing of 149 isolates revealed eight clusters, with the number of SNPs of the largest two clusters ranging between 0 and 116 for cluster 1 (*n* = 49 isolates), and 0 and 27 for cluster 2 (*n* = 36 isolates), among isolates derived between 2009 and 2014.

**Conclusions:**

This study demonstrates a stable low-level endemicity of MREb in two Dutch ICUs with prolonged use of SDD, which was characterized by the persistent presence of two clusters, suggesting incidental clonal transmission.

**Electronic supplementary material:**

The online version of this article (10.1186/s13756-018-0356-7) contains supplementary material, which is available to authorized users.

## Background

*Enterobacter cloacae* are commensal bacteria of the human gut that can inactivate third-generation cephalosporins through overproduction of chromosomally encoded AmpC beta-lactamases, especially during treatment with these antibiotics. Therefore, recommended treatment of infections with these bacteria includes trimethoprim/sulfamethoxazole, fluoroquinolones or carbapenem antibiotics.

The *E. cloacae* complex comprises five species: *E. cloacae*, *Enterobacter asburiae*, *Enterobacter hormaechei*, *Enterobacter kobei*, and *Enterobacter ludwigii*. *E. cloacae* and *E. hormaechei* are the most frequently isolated human isolates [1]. Classification beyond genus level is difficult, and therefore often not performed. Aminoglycoside resistance in *Enterobacter* species is usually attributable to aminoglycoside-modifying enzymes that are often plasmid-encoded but can also be associated with transposable elements [1]. Colistin resistance is mostly caused by chromosomal mutations, most likely inducing changes in the negatively charged surface lipopolysaccharides [2]. Recently, plasmid-mediated resistance encoded by different *mcr* genes has been described [3].

Antibiotics may create a selective pressure leading to detectable carriage with *Enterobacter* species, especially in hospitalized patients (endogenous selection). Although such new detections are often considered acquisition, they do not represent true acquisition events. An increased incidence of healthcare-associated infections or carriage with *E. cloacae* can, therefore, result from endogenous selection, but also from clonal transmission, either or not facilitated by selective antibiotic pressure. Bacterial typing is required to disentangle the relative importance of both acquisition routes.

Selective decontamination of the digestive tract (SDD) is an infection prevention measure consisting of mouthpaste and intragastric suspension containing tobramycin, colistin and amphotericin B, and intravenous cefotaxime during the first days of ICU admission. Selective oropharyngeal decontamination (SOD) only includes the mouthpaste, with the same topical antibiotics. Based on studies in which SDD and SOD were associated with lower ICU mortality, SOD or SDD became standard-of-care in Dutch ICUs [4]. The effect of SDD and SOD on the emergence of colistin resistance among Gram negative bacteria is unknown. In longitudinal studies in Dutch ICUs using SDD this risk was low [5], but outbreaks with colistin-resistant bacteria during SDD have been reported as well [6].

A high incidence of carriage and infection with colistin-resistant *E. cloacae* complex isolates in patients admitted in the intensive care unit (ICU) in a Dutch hospital was noted in June 2014. It was hypothesized that the use of SDD in this ICU since 1990 had contributed to this high incidence, either due to repeated events of endogenous selection or due to incidental introduction of colistin-non-susceptible strains followed by clonal transmission. A detailed retrospective and prospective investigation was performed to investigate the epidemiology of antibiotic-resistant *E. cloacae* complex isolates*.*

## Methods

### Setting

The hospital is a 540-bed secondary care hospital with two ICUs (16 and 6 beds) on physically separated locations. There is intensive exchange of employees and patients between both ICUs. SDD consisting of mouthpaste (500 mg 4 times daily) and intragastric suspension (8 ml 4 times daily) (tobramycin, colistin, and amphotericin B) and intravenous cefotaxime 1000 mg 4 times daily for the first 3 days of ICU admission, was used routinely for patients with an expected length of invasive ventilation of > 24 h and patients receiving enteral tube feeding for an expected length of ICU stay of > 48 h, since 1990. As part of the SDD strategy, screening was performed on admission and twice weekly using throat and rectum swabs. This standard of care was interrupted only between 2004 and 2006 as part of a multicentre trial evaluating the effects of SDD, in which SOD was used for 6 months and neither SDD nor SOD were used for another 6 months [4].

### Data collection

Demographic data, patient location data and data on antibiotic use were extracted from the electronic hospital information system. Data was collected from 1 May 2005 to 1 November 2014. Data on antibiotic prescriptions are available from 1 November 2005, and data on patient location from 1 January 2007. Microbiological data was extracted from the electronic laboratory system, and data were available from 1 May 2005 onwards. Colistin susceptibility was documented since November 2009 and presence of ESBL since January 2011.

### Microbiological methods

Species identification and susceptibility testing of clinical isolates was performed using Vitek 2 (bioMérieux, Marcy-l’Étoile, France). Isolates with elevated MIC for meropenem and/or imipenem were sent to a reference laboratory to test for presence of carbapenemases. Pulsed-field gel electrophoresis (PFGE) had been performed on 96 *Enterobacter* isolates from 62 patients and 8 environmental samples as screening (data not shown).

For isolates selected for whole-genome sequencing (WGS, as described below) species identification was performed by MALDI-TOF (Microflex, Bruker, Germany), antibiotic susceptibility testing was performed by Vitek 2, colistin resistance was additionally determined by Etest (bioMérieux, Marcy-l’Étoile, France), and presence of ESBL by cefepime-clavulanate ESBL Etest (bioMérieux, Marcy-l’Étoile, France), both on Mueller Hinton 2 agar.

### Definitions

Multi-resistant *Enterobacter* species (MREb) was defined as *Enterobacter* isolates with ESBL production and/or tobramycin non-susceptible (MIC> 4 mg/L; EUCAST 2013 [7]) and/or colistin resistant (MIC> 2 mg/L; EUCAST 2013). Furthermore, *Enterobacter* isolates were classified on resistance profile as I) tobramycin non-susceptible *Enterobacter* spp., II) colistin resistant *Enterobacter* spp., or III) ESBL producing *Enterobacter* spp.

### Selection of isolates for whole genome sequencing (WGS)

First, from each patient from whom *Enterobacter* spp. isolates had been stored, isolates in each of the following groups were selected for WGS a) isolates for which PFGE had been performed before, b) blood culture isolates, c) cerebrospinal fluid (CSF) isolates, d) colistin-resistant isolates, e) isolates from ICU. When multiple isolates for one individual patient were available in one of the groups, the final selection per patient was based on 1) location (ICU, ward, outpatient), 2) highest MIC for colistin, tobramycin and ESBL positivity, 3) the most invasive isolate (culture site: blood, CSF, broncho-alveolar lavage, fluid/punctate, wound, urine, sputum, catheter tip, throat, rectum, nose, faeces), 4) culture date (first).

From one patient both colistin-susceptible (*n* = 2) and resistant *E. cloacae* complex isolates (*n* = 1) were selected for WGS. For one patient isolates collected 4 years apart were selected (colistin susceptible, tobramycin susceptible).

As control isolates, which were considered not be linked to the local hospital epidemiology, we included 23 *Enterobacter* isolates; 17 cultured between 2002 and 2014 in another Dutch hospital in the same region, one from Curacao, an island in the Caribbean Sea, and six unrelated isolates.

To test whether colistin resistance had been induced in susceptible isolates that were prevalent in the geographic area, 15 susceptible isolates were also included. These included seven isolates from patients admitted in the same period, six isolates from general practice patients and two isolates from a different hospital in the same city. None of these patients had documented carriage with MREb.

### Whole genome sequencing

Isolates were cultured overnight at 37 °C on blood agar plates. Subsequently one colony was transferred to Lysogeny Broth and incubated overnight at 37 °C. From this culture DNA was isolated using the Ultra Clean Microbial DNA isolation kit (Mo Bio Laboratories, Inc., Carlsbad, CA). Library preparation used the Illumina Nextera XT DNA Sample Preparation kit and sequencing was performed on an Illumina NextSeq with the mid-output 2 × 150 bp kit using paired-ends (Illumina, San Diego, CA).

### Comparative genomics analysis

In total, genomic DNA of 172 *Enterobacter cloacae* complex isolates were sequenced. Sequence reads were first quality-filtered using seqtk with option “trimfq -q 0.01” (version 1.0-r31) [8] and then assembled using SPAdes (version 3.7.0) [9]. Genomes with lengths ranging between 3 Mb and 8 Mb were selected for further analysis, which resulted in a total of 150 genomes. Assembled contigs were annotated using prokka (version 1.12-beta) [10]. Proteins among these strains were first aligned against each other using BLAST+ (version 2.2.31) [11], where length of aligned part must be longer than the half length of both query and reference sequences. Resulting alignment file was used as an input to orthAgogue (version 1.0.3) [12] to find orthologous proteins and orthologous proteins were clustered using mcl (version 12–135) [13]. Genes that are present in all strains (core genes) were concatenated to create a core genome. Next, recombination regions in the core genome were identified using gubbins (version 1.4.9) [14]. Subsequently, all gene sequences in identified recombination regions were discarded and final recombination-filtered core genomes were used to build a phylogenetic tree using FastTree2 (version 2.1.8) with the GTR model and with 100 bootstrap samples [15].

### Statistical analyses

Incidence of acquisition in ICU was calculated per year as number of patients with a positive culture according to one of the classifications, per 1000 patient days at risk. Acquisition was defined as growth of *Enterobacter* spp. from a microbiological culture on ICU-day 3 or thereafter, with a preceding negative culture. Admissions < 48 h were not included in this analysis as these were not considered at risk for acquisition according to this definition. Patient days at risk are the sum of all uncolonized days in ICU of all patients until ICU-discharge or until the day of the first culture with *Enterobacter* spp. Prevalence is calculated as the percentage of colonized patient days divided by the total number of patient days. Sensitivity and specificity of determination of colistin resistance using Vitek were calculated with Etest as reference standard. Data analysis was performed using SPSS version 22 and R version 3.1.2.

## Results

### Microbiology results

From 1 May 2005 to 1 November 2014 *Enterobacter* spp. were detected in 2448 patients in both ICUs, including 1373 MREb isolates from 373 patients. These isolates were derived from rectal/fecal samples (*n* = 105; 28.2%), wounds (*n* = 71; 19.0%), sputum (*n* = 66; 17.7%), urine (*n* = 53; 14.2%), throat/nose (*n* = 48; 12.9%), catheter tips (*n* = 8; 2.1%), broncho-alveolar lavage fluid (*n* = 7; 1.9%), blood (*n* = 6; 1.6%) and other material (*n* = 9; 2.4%). Among the MREb isolates proportions of non-susceptibility were 68.8% for colistin (141 of 205 tested), 89.3% for tobramycin (333 of 373 tested) and 71.2% had ESBL production (111 of 156 tested) (Table 1). Of the MREb isolates, 82.3% was susceptible to either ciprofloxacin or trimethoprim/sulfamethoxazole, 99.1% to ciprofloxacin, trimethoprim/sulfamethoxazole or meropenem, and 99.5% to ciprofloxacin, trimethoprim/sulfamethoxazole, meropenem or colistin. Twenty-two of 373 patients with MREb had isolates with elevated MICs for imipenem and/or meropenem. PCR testing for carbapenemase-producing genes was performed for isolates of 12 patients and New Delhi metallo-beta-lactamase (NDM) was detected in one isolate. This patient had been hospitalized for 1 day in 2011, after a recent return from Mecca, Saudi Arabia.

**Table 1:** Antibiotic resistance in patients with resistant *E. cloacae* and susceptible *E. cloacae*

Antibiotic (combination)	Susceptible *E. cloacae* (*n* = 2075 unique patients, 3030 isolates)		Multi-resistant *E. cloacae* (*n* = 373 patients, 1373 isolates)		Selected for WGS (*n* = 112 isolates)
	Patients (n) tested	% non-susceptible / positive^a^	Patients (n) tested	% non- susceptible / positive^a^	% non- susceptible / positive^a^
ciprofloxacin	2075	1.3	373	42.9	28.6
colistin	1122	0	205	68.8	78.4^b^
gentamicin	2075	0.2	373	82.8	86.6
imipenem	2070	0.2	373	5.6	6.3
meropenem	2073	0.1	373	4.8	6.3
tobramycin	2075	0	373	89.3	87.5
trimethoprim/ sulfamethoxazole	2075	5.5	373	34.6	86.6
ESBL production	873	0	156	71.2	72.3
CIP or SXT	2075	0.6	373	17.7	5.4
CIP or SXT or MEM	2064	0	349	0.9	0
CIP or SXT or MEM or CST	1118	0	187	0.5	0

*CIP* ciprofloxacin, *CST* colistin, *ESBL* extended-spectrum beta-lactamase, *MEM* meropenem, *n* number, *SXT* trimethoprim/ sulfamethoxazole, *WGS* whole-genome sequencing

^a^% non-susceptible for antibiotics or % ESBL positive

^b^111/112 isolates tested

Number of resistant *E. cloacae* isolates tested: ciprofloxacin: *n* = 1373, colistin: *n* = 784, gentamicin: *n* = 1373, imipenem: *n* = 1373, meropenem: *n* = 1371, tobramycin: *n* = 1373, trimethoprim/ sulfamethoxazole: *n* = 1372. Number of susceptible *E. cloacae* isolates tested: ciprofloxacin: *n* = 3030, colistin: *n* = 1613, gentamicin: *n* = 3030, imipenem: *n* = 3024, meropenem: *n* = 3028, tobramycin: *n* = 3030, trimethoprim/ sulfamethoxazole: *n* = 3030

From 1 January 2005 onwards there were 97 *E. cloacae* bacteremia episodes in 96 patients, and 13 isolates (13.5%) were non-susceptible to tobramycin. Since 2009, though, a tobramycin and colistin non-susceptible isolate was obtained from only one of 42 patients (2.4%) with *E. cloacae* bacteremia.

Comparison of colistin susceptibility testing by Vitek and Etest of 100 isolates (86 being resistant) yielded sensitivity and specificity of 100% (Additional file 1: Table S1).

### Incidence and prevalence of MREb

There were (together in both ICUs) 12,680 ICU admissions of 10,741 patients between 1 January 2007 and 31 October 2014; 3506 admissions (3210 patients) had an ICU-stay > = 48 h. On admission prevalence of MREb was 1.7% (61/3506; 61 admissions of 49 patients), and 154 patients acquired MREb during ICU-stay (Table 2). In 73 patients carriage or infection was detected after ICU discharge. Of the 373 patients with MREb, 146 could not be related to stay in either of the two ICUs. For 102 of these 146 patients data collection was incomplete, as ICU admission data were available from 2007 onwards.

**Table 2:** Incidence of resistant *E. cloacae* in ICU 1 and 2

	Selection	ICU 1		ICU 2		Period
		All admissions	Admissions > 48 h	All admissions	Admissions > 48 h	
Admissions (n)	All	9418	2731	3624	884	1 Jan 2007–1 Nov 2014
Patients (n)		8073	2301	3257	836	1 Jan 2007–1 Nov 2014
On admission	MREb	58^a^		25^a^		1 Jan 2007–1 Nov 2014
Acquisitions (n)		134		20		1 Jan 2007–1 Nov 2014
Mean incidence (acquisitions/DAR)	MREb	4.60 (69/14977)		1.85 (6/3248)		1 Jan 2011–1 Nov 2014
	Tobramycin I or R	4.95 (130/26265)		2.74 (20/7312)		1 Jan 2007–1 Nov 2014
	Colistin R	5.02 (81/16139)		2.28 (10/4386)		1 Jan 2010–1 Nov 2014
	ESBL production	4.64 (58/12489)		1.88 (6/3200)		1 Jan 2011–1 Nov 2014

*DAR* days at risk, *ESBL* extended-spectrum beta-lactamase, *I* intermediate, *ICU* intensive care unit, *MREb* extended-spectrum beta-lactamase production and/or tobramycin non-susceptible and/or colistin-resistant, *n* number, *R* resistant. Colistin susceptibility was documented since December 2009 and presence of ESBL since January 2011

^a^These numbers include patients transmitted between the two ICUs. In total in 61 ICU admissions MREb was detected before or on admission

**Fig. 1:**
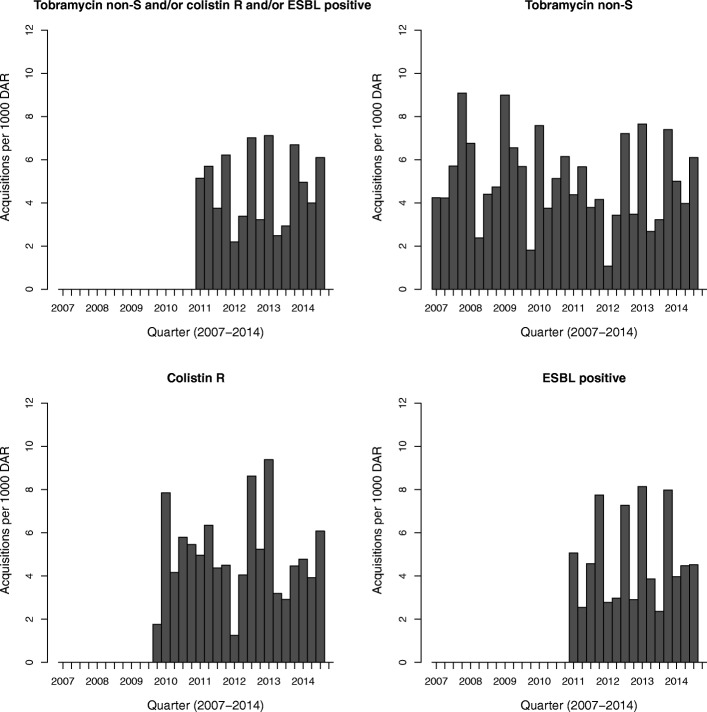
Incidence of resistant *E. cloacae* on ICU 1. ESBL: extended-spectrum beta-lactamase, non-S: non-susceptible, R: resistant

**Fig. 2:**
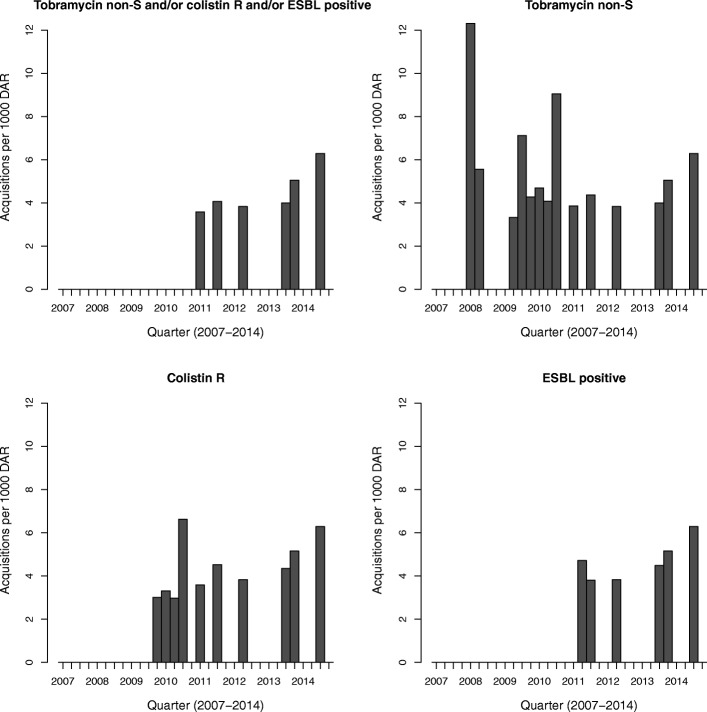
Incidence of resistant *E. cloacae* on ICU 2. ESBL: extended-spectrum beta-lactamase, non-S: non-susceptible, R: resistant

Incidence rates of MREb acquisition per 3 months from 2007 to 2014 are shown in Figs. 1 and 2. MREb acquisition rates ranged from 4 to 7 per 1000 days at risk, with no discernable trend in time. Similar ranges of acquisition were observed for the three individual resistance markers.

**Fig. 3:**
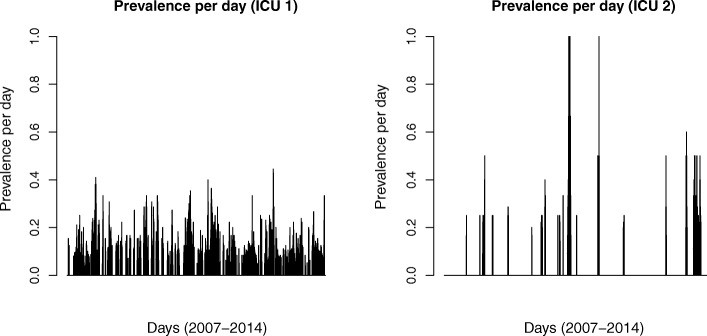
Daily prevalence of ESBL producing and/or tobramycin non-susceptible and/or colistin-resistant *E. cloacae*, in ICU (ICU 1 and ICU 2)

MREb was regularly present in ICU 1, with at least one patient carrying MREb being present in 53.8% of all days, and a monthly mean prevalence of 7.0% (19.6 per 282 patient days). MREb was incidentally present in ICU 2, with 8.9% of days at least one patient carrying MREb admitted, and monthly mean prevalence of 3.1% (2.5 per 80 patient days; Fig. 3). There was no discernable trend in prevalence in time, with more variation in prevalence in ICU 2 because of the lower number of patients admitted at each moment.

From 2007 onwards, up to 81 patients had not been admitted to ICU before their first positive culture with resistant *E. cloacae.*

### Acquisition of colistin resistance

A change in colistin susceptibility (from susceptible to resistant) of *E. cloacae* detected in screening cultures was observed in three patients with rectal colonization and in two other patients with respiratory colonization, corresponding to conversion rates of 0.20 and 0.13 conversions per 1000 patient days of *E. cloacae* carriage. All five patients received SDD.

### Whole genome sequencing

**Fig. 4:**
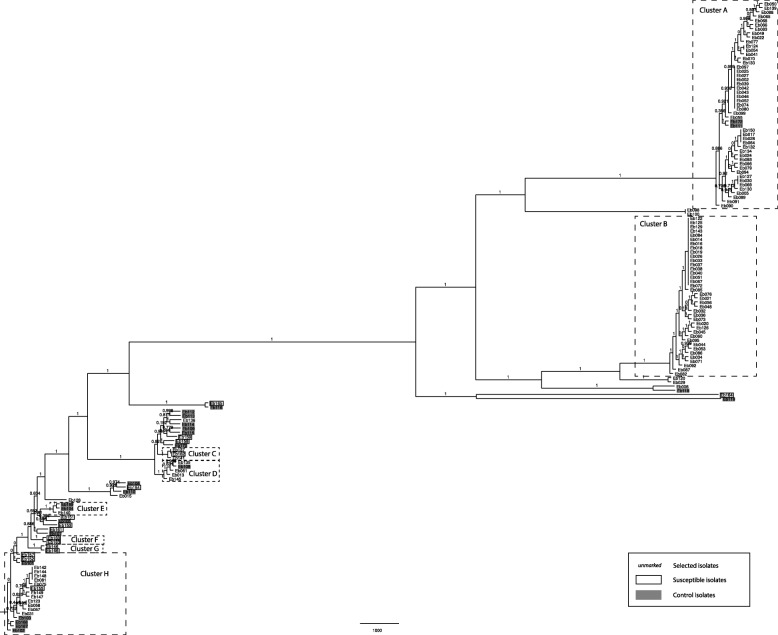
Whole genome sequencing of selected *E. cloacae* isolates (full phylogenetic tree)

Of 362 stored *E. cloacae* isolates, 112 isolates from 96 patients were selected for WGS. Susceptibility profiles of these isolates are shown in Table 1. The phylogenetic tree was built on 112 MREb isolates, 15 susceptible isolates, and 23 control isolates (Fig. 4). WGS revealed eight clusters, of 49, 38, 3, 5, 3, 2, 2 and 19 isolates respectively, with the number of SNPs of the largest two clusters ranging between 0 and 116 for cluster A (*n* = 49 isolates), and 0 and 27 for cluster B (*n* = 38 isolates). The oldest cluster A isolate was identified in June 2009 and the youngest in May 2014. Cluster B isolates were derived between December 2009 and August 2014.

There was a high level of concordance in antibiotic susceptibility within clusters, except for ESBL-production (Table 3). In clusters A and B, the majority of isolates were non-susceptible to colistin (46 of 47 isolates tested and 37 of 38 isolates tested), and non-susceptible to tobramycin (46 of 47 isolates tested and 38 of 38 isolates tested). Seventy-two were ESBL-producers (41 of 45 isolates tested and 31 of 33 isolates tested). All cluster C to G isolates are colistin and tobramycine-susceptible. All but one cluster H isolates are colistin susceptible and tobramycine resistant. This isolate is the only isolate outside of clusters A and B that is both tobramycin and colistin non-susceptible. In the one patient with three isolates tested, two non-MREb bacteremia isolates (Eb098 and Eb100) were clearly distinct from the MREb isolate (Eb099) in cluster A.

**Table 3:** Characteristics of selected *E. cloacae* isolates

Cluster	Number of isolates	Location of patients located in hospital 1 or 2 at time of culture	Period of detection	Resistance (n/n tested)		
				Colistin	Tobramycin	ESBL
A	49	Intensive care unit (34 patients ICU 1; 5 patients ICU 2)	June 2009 – May 2014	non-S (46/47)	non-S (46/47)	positive (41/45)
B	38	Intensive care unit (29 patients ICU 1; 5 patients ICU 2)	December 2009 – August 2014	non-S (37/38)	non-S (38/38)	positive (31/33)
C	3	Ward (2 patients hospital 1; 1 patient hospital 2)	June 2013 – February 2014	S (3/3)	S (3/3)	negative (2/3)
D	5	Intensive care unit (3 patients ICU 1)	September 2009 – May 2012	S (4/4)	S (4/4)	positive (4/4)
E	3	Ward (1 patient hospital 1)	January 2014 – January 2014	S (1/1)	S (1/1)	positive (1/1)
F	2	Ward/outpatient (2 patients hospital 1)	July 2013 – August 2014	S (2/2)	S (2/2)	negative (2/2)
G	2	Ward (1 patient hospital 1)	February 2012 – April 2012	S (2/2)	S (2/2)	positive (1/2)
H	19	Intensive care unit (6 patients hospital 1), ward (5 patients hospital 1)	April 2010 – July 2014	S (13/14)	non-S (13/14)	positive (7/14)

*ESBL* extended-spectrum beta-lactamase, *ICU* intensive care unit, *n* number, *non-S* non-susceptible, *S* susceptible

## Discussion

This study demonstrates a persistently stable low-level endemicity of *Enterobacter* species resistant to colistin and/or tobramycin in a Dutch ICU that used SDD since 1999. The incidence of acquisition of resistant *E. cloacae* was 4.61 and 1.86 per 1000 admission days for ICU 1 and 2 respectively, and the mean prevalence was 7 and 3%, respectively. Although 69% of the resistant isolates were resistant to colistin, susceptibility to either one of the preferred antibiotics for infections caused by *Enterobacter* species, such as co-trimoxazole, ciprofloxacin or meropenem, was 99%. Carbapenemase production was demonstrated in one resistant *E. cloacae* isolate, which appeared to be introduced from abroad. Therefore, resistance in these *Enterobacter* species did not reduce antibiotic treatment options. WGS revealed two large clusters spanning a prolonged time period, strongly suggesting incidental clonal transmission, sufficient to maintain low-level endemicity, which probably is higher than in most other Dutch ICUs.

Evaluation of long-term trends in antibiotic resistance in ICU isolates in June 2014 led to the recognition of the presence of colistin-resistant *Enterobacter* species, and initiated the formation of an outbreak management team, including medical microbiologists, infection prevention specialists, intensivists, ICU nurses, infectiologists, and facilitary services. A variety of measures were taken including reinforcement of existing infection prevention measures, including hand hygiene precautions and isolation protocols, replacement of dated equipment, daily infection prevention audits and renovation of some spaces. On 1 September 2014 SDD was replaced by SOD.

The investigation described here started in January 2015 (after all measures had been implemented). The focus of investigation was on the ICU where screening cultures had been obtained routinely as part of SDD. As such, the likelihood of detecting such isolates outside the ICUs is lower. Yet, from 2007 onwards, up to 81 patients had not been admitted to ICU before their first positive culture with resistant *E. cloacae*, suggesting that transmission was not limited to the ICUs.

In this study, colistin susceptibility testing as performed by Vitek appeared adequate in distinguishing resistant from susceptible isolates using Etest as a comparator. It should be noted that gradient tests have recently been tested to generally underestimate MICs, resulting in false susceptible results [16]_,_ thus misclassification in our study cannot be ruled out. Yet, there were only a limited number of isolates with an MIC in the intermediate range.

The possible effects of colistin use in SDD could not be disentangled, as SDD was administered to all eligible patients admitted to the ICU, precluding a comparison with control patients. The conversion rate from carriage of colistin-susceptible to resistant *E. cloacae* (0.20 and 0.13 conversions per 1000 patient days) is lower than previously reported in ICUs using SDD [5], even though we might have overestimated true conversion in these patients as susceptible isolates of these patients were not routinely stored. Previously, estimated conversion rates for colistin in *Enterobacter* spp., *Escherichia coli* or *Klebsiella* spp*.* during SDD in rectal isolates were 1.0 and 0.7 per 1000 patient days in two cohorts [5]. For respiratory isolates conversion rates in *Acinetobacter* spp., *Pseudomonas* spp., *Enterobacter* spp., *E. coli* or *Klebsiella* spp*.* during SDD were 0.7 per 1000 patient days. In that study 1 of 17 and 3 of 9 conversions in intestinal colonization, and 4 of 12 conversions in respiratory colonization (standard care, SOD and SDD combined) were in *Enterobacter* species [5].

WGS provided essential information for interpreting the molecular epidemiology of *Enterobacter* spp. in these ICUs. Plasmids do not belong to the core genome, and are not taken into account in the analysis of WGS. Plasmid-based colistin resistance genes (*mcr*-1 to 4) were not detected in any of the sequenced isolates (data not shown). The relatively low correlation between clustering and presence of ESBL may have resulted from horizontal transfer of plasmids encoding ESBLs. There is limited data on the number of expected SNPs in clonal transmission. In a set of presumed outbreak isolates based on identical PFGE patterns from two patients isolates had less than 22 SNPs, while isolates from a third patient were distinct with a distance greater than 150 SNPs [17]. It was concluded that the third patient was unlikely to have been associated with a transmission event involving one of the other two patients. We found two clusters with up to 126 SNPs.

## Conclusions

A high incidence of carriage and infection with MREb isolates in patients admitted in the ICU in a Dutch hospital was noted in June 2014. It was hypothesized that the use of SDD in this ICU since 1990 had contributed to this high incidence, either due to repeated events of endogenous selection or due to incidental introduction of MREb strains followed by clonal transmission. These data demonstrate a persistent low prevalence of carriage with MREb, most probably maintained by incidental cross-transmission events and repeated (sporadic) introductions of carriers. These findings are compatible with a scenario in which SDD caused repeated events of endogenous selection, but in which existing infection control measures prevented uncontrolled spread, as well as with a scenario in which SDD prevented higher frequencies of carriage and subsequent uncontrolled spread due to insufficient infection control measures. Our findings also demonstrate that SDD must be accompanied by appropriate microbiological surveillance and good adherence to infection control measures in the unit.

## Additional file

Additional file 1: **Table S1.** Colistin minimum inhibitory concentrations (MIC) as determined by Vitek and Etest for 100 *Enterobacter* isolates. (DOCX 15 kb)

## Abbreviations

CSF: Cerebrospinal fluid
ESBL: Extended-spectrum beta-lactamase
ICU: Intensive care unit
MIC: Minimum inhibitory concentration
MREb: Multi-resistant *Enterobacter cloacae*
NDM: New Delhi metallo-beta-lactamase
PFGE: Pulsed-field gel electrophoresis
SDD: Selective decontamination of the digestive tract
SNP: Single mucleotide polymorphism
SOD: Selective oropharyngeal decontamination
WGS: Whole genome sequencing
