# Supplementary material for: Dynamics of colistin and tobramycin resistance among Enterobacter cloacae during prolonged use of selective decontamination of the digestive tract
Source: Antimicrob Resist Infect Control. 2018 May 22;7:67. doi: 10.1186/s13756-018-0356-7 (PMC5964641; doi:10.1186/s13756-018-0356-7)
Supplement: Supplementary file 1 — Table S1. Colistin minimum inhibitory concentrations (MIC) as determined by Vitek and Etest for 100 Enterobacter isolates. (DOCX 15 kb) [file 13756_2018_356_MOESM1_ESM.docx]

**Additional file 1**

**Table S1:** Colistin minimum inhibitory concentrations (MIC) as determined by Vitek and Etest for 100 *Enterobacter* isolates.

| **MIC (mg/mL, Etest)** | **MIC (mg/mL, Vitek)** | | | |
| --- | --- | --- | --- | --- |
|  | **<=0.5** | **4** | **8** | **>=16** |
| **0.19** | 1 | 0 | 0 | 0 |
| **0.25** | 1 | 0 | 0 | 0 |
| **0.38** | 8 | 0 | 0 | 0 |
| **0.5** | 4 | 0 | 0 | 0 |
| **24** | 0 | 1 | 0 | 0 |
| **48** | 0 | 0 | 0 | 4 |
| **64** | 0 | 0 | 1 | 2 |
| **96** | 0 | 0 | 0 | 2 |
| **128** | 0 | 0 | 0 | 1 |
| **192** | 0 | 0 | 0 | 3 |
| **256** | 0 | 0 | 0 | 17 |
| **384** | 0 | 0 | 0 | 9 |
| **512** | 0 | 0 | 0 | 12 |
| **768** | 0 | 0 | 0 | 5 |
| **>1024** | 0 | 0 | 0 | 29 |

MIC: minimum inhibitory concentration.
